# A Clinical Prediction Tool for Extended-Spectrum Cephalosporin Resistance in Community-Onset Enterobacterales Urinary Tract Infection

**DOI:** 10.1093/ofid/ofz164

**Published:** 2019-03-14

**Authors:** Erica J Weinstein, Jennifer H Han, Ebbing Lautenbach, Irving Nachamkin, Charles Garrigan, Warren B Bilker, Lois Dankwa, Mary Wheeler, Pam Tolomeo, Judith A Anesi, CDC Prevention Epicenters Program

**Affiliations:** 1 Division of Infectious Diseases, Department of Medicine; 2 Center for Clinical Epidemiology and Biostatistics; 3 Department of Biostatistics, Epidemiology and Informatics; 4 Department of Pathology and Laboratory Medicine, Perelman School of Medicine, University of Pennsylvania, Philadelphia, Pennsylvania, USA

**Keywords:** community-onset, Enterobacterales, extended-spectrum cephalosporin resistance, prediction tool, urinary tract infection

## Abstract

**Background:**

Bacterial resistance to first line antibiotics used to treat community-onset urinary tract infections (UTIs) continues to increase. We sought to create a clinical prediction tool for community-onset UTIs due to extended-spectrum cephalosporin-resistant (ESC-R) Enterobacterales (formerly Enterobacteriaceae, EB).

**Methods:**

A case-control study was performed. The source population included patients presenting to an emergency department (ED) or outpatient practice with an EB UTI between 2010 and 2013. Case patients had ESC-R EB UTIs. Control patients had ESC-susceptible EB UTIs and were matched to cases 1:1 on study year. Multivariable conditional logistic regression was performed to develop the predictive model by maximizing the area under the receiver-operating curve (AUC). Internal validation was performed via bootstrapping.

**Results:**

A total of 302 patients with a community-onset EB UTI were included, with 151 cases and 151 controls. After multivariable analysis, we found that presentation with an ESC-R EB community-onset UTI could be predicted by the following: (1) a history of malignancy; (2) a history of diabetes; (3) recent skilled nursing facility or hospital stay; (4) recent trimethoprim-sulfamethoxazole exposure; and (5) pyelonephritis at the time of presentation (AUC 0.73, Hosmer-Lemeshow goodness-of-fit *P* value 0.23). With this model, each covariate confers a single point, and a patient with ≥ 2 points is considered high risk for ESC-R EB (sensitivity 80%, specificity 54%). The adjusted AUC after bootstrapping was 0.71.

**Conclusions:**

Community-onset ESC-R EB UTI can be predicted using the proposed scoring system, which can help guide diagnostic and therapeutic interventions.

## Teaser

Bacterial resistance continues to increase in the community. We found that community-onset urinary tract infections due to extended-spectrum cephalosporin-resistant Enterobacterales (formerly Enterobacteriaceae) could be predicted with knowledge of 5 clinical factors.

Urinary tract infections (UTIs) are one of the most common causes for antibiotic prescription in the outpatient setting [1]. The majority of UTIs are caused by members of the Enterobacterales (EB) family (formerly Enterobacteriaceae family), particularly *Escherichia coli* [2]. The Infectious Diseases Society of America recommends that in cases of uncomplicated UTI, providers may treat without obtaining a urine culture [2]. Further, with pyelonephritis and complicated UTIs, they recommend that clinicians should begin empiric antimicrobial therapy while awaiting culture results [2].

Choosing the correct empiric antibiotic for UTI has become more challenging as the proportion of EB infections, including community-onset infections, that exhibit extended-spectrum cephalosporin resistance (ESC-R) has increased [3–8]. For example, over the 3 year period between 2000 and 2003, a Spanish hospital reported a 3-fold increase in community-onset UTIs caused by extended-spectrum β-lactamase (ESBL)-producing *E. coli* [6]. In the US during this same time period, ESBL-producing EB were less common in the community; over the last decade, however, their presence has been increasingly noted [9]. Recent studies in the US have reported that 36–83% of ESBL-producing *E. coli* infections in the US had a community onset [10, 11]. These community-onset ESC-R EB UTIs are associated with increased patient morbidity and healthcare costs and thus represent a significant, and growing, clinical problem [12–18].

Clinical prediction tools can be helpful for identifying patients at higher risk for a multidrug-resistant (MDR) infection, which in turn can help guide diagnostic evaluation and empiric therapies. Although prediction tools to identify patients who are admitted to an emergency department (ED) or hospital with bacteremia secondary to ESBL-EB organisms have been developed [19], to date there are no published clinical prediction tools focused on identification of community-onset ESC-R EB UTIs, particularly those without bacteremia. A clinical prediction tool for such patients presenting to an outpatient clinic or ED with a community-onset UTI would be highly valuable for determining (1) which patients require a urine culture with an uncomplicated UTI syndrome and (2) the appropriate empiric antibiotics for those with complicated UTI or pyelonephritis. As a result, the objective of this study was to create a predictive tool that categorizes patients as high or low risk for an ESC-R EB as the etiology of a community-onset UTI at the time of the first clinical evaluation for UTI.

## MATERIALS AND METHODS

### Study Design and Setting

A case-control study was performed at 2 EDs and a network of ambulatory practices within the University of Pennsylvania Health System (UPHS) [18]. More specifically, it included the following: (1) the ED at the Hospital of the University of Pennsylvania (HUP), a 776-bed quaternary care medical center; (2) the ED at Penn Presbyterian Medical Center (PPMC), a 331-bed academic medical center, and (3) a network of 246 primary care physicians at community and hospital-based practices.

### Study Population

The initial source population was composed of all patients presenting to an ED or outpatient practice who had a urine culture positive for an EB organism between December 21, 2010 and April 22, 2013. Potentially eligible patients were identified through the HUP Clinical Microbiology Laboratory, which processes all cultures from HUP and PPMC, as well as >90% of urine cultures from UPHS outpatient practices. A patient was designated as having a community-onset urine culture if it was obtained in the ED, outpatient practices, or within 72 hours of hospital admission. Subsequently, patients were excluded if they were <18 years, expired during the follow-up period, were a long-term care facility resident, or had a physician who did not provide consent. The remaining subjects were eligible and were approached for consent. Those with an ESC-R EB organism on urine culture were approached first, and then a random selection of those with an ESC-S EB organism on urine culture were approached in an equal number as those with ESC-R EB. After consenting, only patients with a true UTI were included as we sought to identify predictors of ESC-R EB UTIs rather than urinary colonization. A urine culture was considered indicative of an infection based on the Centers for Disease Control and Prevention (CDC) National Healthcare Safety Network (NHSN) criteria [20], which was determined via medical record review performed by an infectious diseases-trained physician (J.H.H.).

Case patients were defined as those with an EB UTI demonstrating resistance to an ESC (ie, ceftriaxone or cefotaxime minimum inhibitory concentration [MIC] >1mg/L) according to Clinical and Laboratory Standards Institute (CLSI) criteria [21]. Control patients were those who had a UTI with ESC-susceptible EB during the study period (ie, ceftriaxone and cefotaxime MICs ≤ 1 mg/L). Control patients were randomly selected from among all patients with ESC-susceptible EB UTIs using a computerized random number generator and were matched with exposed patients in a 1:1 ratio based on study year.

Each patient was included as a subject only once, and if an EB was isolated on multiple occasions in the same patient, only the first episode of infection was considered in these analyses. The institutional review board of the University of Pennsylvania approved the study.

### Data Collection

Data on case and control patients were abstracted from the UPHS electronic medical record. Information was collected on demographics (eg, age, gender, and race), comorbidities (eg, diabetes, malignancy, and chronic kidney disease), urologic disorders (eg, prior UTIs, urinary catheters, and prostate disease), skilled nursing facility or hospital stays in the prior 6 months, and culture location (ED, inpatient, or outpatient). All inpatient and outpatient antibiotic therapy in the 6 months before the index UTI was recorded.

### Susceptibility Testing of Enterobacterales Isolates

Susceptibility testing of EB isolates was performed at the HUP Clinical Microbiology Laboratory. All isolates identified from study subjects were tested as part of routine care for susceptibility to antibiotics using the semi-automated Vitek 2 identification and susceptibility system (bioMerieux, Inc., Durham, NC). Updated MIC breakpoints for ceftriaxone and cefotaxime were used without confirmatory ESBL testing according to CLSI guidelines [21].

### Statistical Analysis

Case and control patients were characterized by potential predictors, including demographics, comorbidities, and prior antibiotic use. For these paired data, continuous variables were compared using the Wilcoxon signed rank test, and categorical variables were compared using the McNemar test. A predictive model was developed using conditional multivariable logistic regression. First, bivariable analyses were performed to determine the relationship between each predictor variable and the outcome of interest (ESC-R EB UTI). A multivariable conditional logistic regression model was then developed to determine the independent predictors using a forward stepwise procedure to maximize the area under the receiver operating curve (AUC) [22, 23]; covariates were added to the model until the AUC was improved by less than 2%. A simplified scoring system was then developed based on the magnitude of the coefficient for each variable in the final predictive model. The internal validity of the tool was assessed with the following: (1) calibration by calculating the Hosmer-Lemeshow statistic [24]; (2) discrimination by calculating the AUC, sensitivity, specificity, positive predictive value, and negative predictive value; and (3) the bootstrapping technique [25].

## RESULTS

### Study Population

There were 2009 unique subjects who grew an EB species on a urine culture from an outpatient visit, ED visit, or within 72 hours of hospital admission during the study period. After applying exclusion criteria, there were 887 subjects who were eligible. Of these 887 potential subjects, 574 (65%) consented to participate in the study. Of these, 151 had an ESC-R EB on urine culture that was consistent with true UTI (rather than colonization) and were thus the final “case” group. One hundred fifty-one patients with community-onset UTI due to an ESC-susceptible EB were then matched to the exposed patients based on study year and comprised the final “control” group.

Among the entire study cohort of 302 patients, the median age was 56 years (interquartile range [IQR], 37–68), and 62 (21%) were men. Two hundred and seventeen (72%) patients presented to an outpatient practice, while 85 (28%) patients presented to the ED. In the control group, 119 (79%) were enrolled from the outpatient clinic and 29 (19%) were enrolled from an ED. In the case group, 89 (59%) were enrolled from the outpatient clinic and 54 (36%) were enrolled from an ED. The most common pathogens isolated were *Escherichia coli* (76%), *Klebsiella* species (13%), and *Enterobacter* species (9%); there were no significant differences in the distribution of organisms between the cases and controls. Forty-three (14%) patients required admission to a hospital. Sixty-two (21%) had pyelonephritis on presentation, and 9 (3%) were bacteremic on presentation with the UTI. Details of the baseline characteristics, comorbidities, and recent antibiotic exposures among the cases and controls have been published previously [18].

### Predictive Model for Community-Onset ESC-R EB UTI

On bivariable analysis (Table 1), we found several factors were predictive of community-onset ESC-R EB UTIs. The factors that were most predictive (ie, had the highest AUC values on bivariable analysis) included: advanced age (AUC 0.64), requirement for hospital admission (AUC 0.60), pyelonephritis at the time of presentation (AUC 0.59), and hospitalization or skilled nursing facility (SNF) stay in the prior 6 months (AUC 0.60).

**Table 1.** Bivariable Analysis of Predictors of Community-Onset Urinary Tract Infection due to an Extended-Spectrum Cephalosporin-Resistant Enterobacterales Organism

Baseline characteristic	AUC	OR (95% CI)	*P* value
Age	0.64	1.03 (1.01–1.04)	<.01
Female gender	0.58	0.37 (0.20–0.70)	<.01
Nonwhite race	0.51	0.92 (0.59–1.45)	.73
Urine culture taken in the ED or within 48 hours of hospital admission	0.60	2.76 (1.59–4.81)	<.01
Urinary catheter at baseline	0.56	4.00 (1.64–9.79)	<.01
Prior UTI^a^	0.53	1.22 (0.79–1.90)	.37
Prior surgery^a^	0.55	1.79 (1.02–3.14)	.04
Pyelonephritis at time of diagnosis	0.59	3.12 (1.65–6.06)	<.01
Recent SNF stay^a^	0.52	4.00 (0.85–18.84)	.08
Recent hospitalization^a^	0.60	2.55 (1.52–4.28)	<.01
Recent SNF stay or hospitalization^a^	0.60	2.55 (1.52–4.28)	<.01
**Comorbidities**			
Malignancy	0.56	3.83 (1.56–9.41)	<.01
Diabetes mellitus	0.56	2.7 (1.31–5.58)	.01
Hemodialysis	0.51	5.00 (0.58–42.80)	.14
Liver disease^b^	0.52	8.00 (1.00–63.96)	.05
Pulmonary disease^c^	0.54	1.80 (0.96–3.38)	.07
Renal transplant	0.52	2.17 (0.82–5.70)	.12
**Antibiotic use** ^**a**^			
Aminoglycoside	0.51	3.00 (0.31–28.84)	.34
Amoxicillin or ampicillin	0.51	2.33 (0.60–9.02)	.22
Amoxicillin/clavulanic acid or ampicillin/sulbactam	0.52	2.29 (0.81–6.48)	.12
Azithromycin	0.51	0.50 (0.09–2.73)	.42
Clindamycin	0.50	0.80 (0.21–2.98)	.74
Doxycycline	0.51	1.67 (0.40–6.97)	.48
Extended-spectrum cephalosporin	0.55	4.75 (1.62–13.96)	.01
Extended-spectrum penicillin	0.52	2.75 (0.88–8.64)	.08
First-generation cephalosporin	0.53	2.43 (1.01–5.86)	.05
Fluoroquinolones	0.51	1.08 (0.69–1.69)	.73
Fosfomycin	0.50	2.00 (0.18–22.06)	.57
Meropenem	0.51	5.00 (0.58–42.80)	.14
Metronidazole	0.51	2.00 (0.50–8.00)	.33
Nitrofurantoin	0.51	1.08 (0.62–1.91)	.77
Trimethoprim-sulfamethoxazole	0.56	2.06 (1.14–3.75)	.02
Vancomycin	0.53	10.00 (1.28–78.12)	.03

Abbreviations: AUC, area under the curve; CI; confidence interval; ED, emergency department; OR, odds ratio; SNF, skilled nursing facility; UTI, urinary tract infection.

^a^In the preceding 6 months.

^b^Hepatitis or cirrhosis.

^c^Chronic obstructive pulmonary disease or chronic bronchitis.

On multivariable analysis (Table 2), we found that the most parsimonious predictive model included: diabetes mellitus, malignancy, SNF stay or hospitalization within the prior 6 months, pyelonephritis at the time of diagnosis, and exposure to trimethoprim-sulfamethoxazole in the prior 6 months (AUC 0.73). Because each covariate had a similar regression coefficient, a scoring system was developed in which each covariate conferred a single point to the patient. The clinical prediction score is a summation of scores from the presence of each independent predictor variable. The possible score ranges from 0 to 5.

**Table 2.** Final Multivariable Predictive Model of Community-Onset Urinary Tract Infection due to an Extended-Spectrum Cephalosporin-Resistant Enterobacterales Organism, Including Scoring Points

Variable	AUC	aOR (95% CI)	*P* value	Scoring points
Recent SNF or hospital stay^a^	0.60	2.55 (1.52–4.28)	<.01	1
Pyelonephritis at time of presentation	0.59	3.12 (1.65–6.06)	<.01	1
History of malignancy	0.56	3.83 (1.56–9.41)	<.01	1
History of diabetes mellitus	0.56	2.70 (1.31–5.58)	.01	1
Recent trimethoprim-sulfamethoxazole exposure^a^	0.56	2.06 (1.14–3.75)	.02	1

Abbreviations: aOR, adjusted odds ratio; AUC, area under the curve; CI, confidence interval; SNF, skilled nursing facility.

^a^In the preceding 6 months.

**Figure 1.**
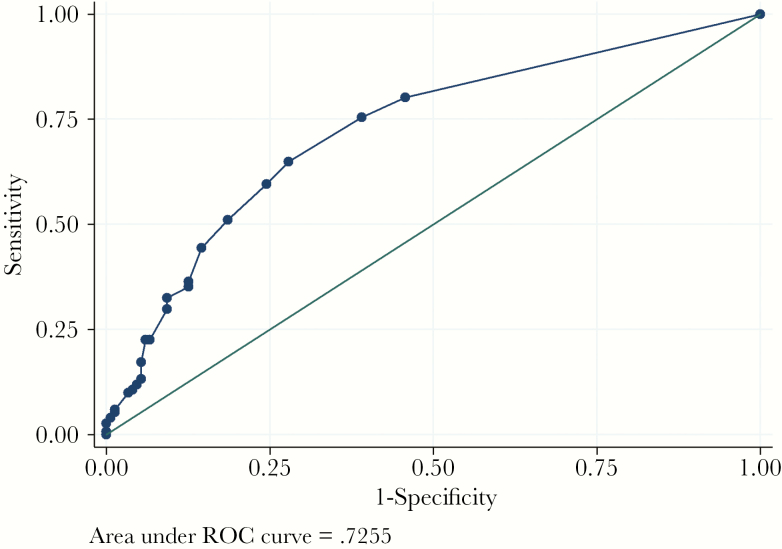
ROC, receiver operating curve.

This clinical prediction rule performed well with internal validation. It showed good calibration (Hosmer-Lemeshow test *P*-value 0.23) and good discrimination (AUC 0.73). The overoptimism-adjusted AUC was 0.71. The receiver operating curve is shown in Figure 1. At the cutoff score of 2 or greater (Table 3), the clinical prediction score demonstrated 80.1% sensitivity, 54.3% specificity, as well as a positive predictive value of 63.7%, and a negative predictive value of 73.2% to identify ESC-R EB UTI.

**Table 3.** Sensitivity and Specificity for Each Cutoff Value of the Simplified Scoring System

Score cutoff value	Sensitivity	Specificity
≥0	100.0%	0.0%
≥1	100.0%	0.0%
≥2	80.1%	54.3%
≥3	44.4%	85.4%
≥4	11.3%	96.0%
≥5	0.0%	100.0%

## DISCUSSION

In this study, we developed a novel clinical prediction tool for community-onset UTI. We found that patients presenting with a community-onset EB UTI are high risk for an ESC-R EB as the etiology if the patient had 2 or more of the following at baseline: a SNF or hospital stay within the prior 6 months, trimethoprim-sulfamethoxazole exposure in the prior 6 months, a history of malignancy, a history of diabetes, or pyelonephritis at the time of UTI diagnosis. The scoring system is simple and requires information on only 5 predictive factors, all of which are clinically based and can be identified without the need for laboratory work or additional testing.

Despite its simplicity, our predictive tool had an AUC >0.70 and sensitivity of 80%. Because an AUC of >0.70 has been described in prior studies as a standard for acceptable predictive ability [26–28], we sought to develop the most parsimonious model that achieved an AUC in that range. Our proposed prediction tool was able to achieve that goal with the inclusion of only 5 clinical factors, making it both acceptably predictive of the outcome and simple enough to be useful in clinical practice. Further, we opted to prioritize sensitivity over specificity, as we believe the most useful clinical application would be in ruling out the possibility of a MDR infection.

There have been prior studies that have developed clinical prediction tools to help identify patients who are high risk for ESBL-EB UTIs. Garcia-Tello *et al* created a normogram to predict the probability of ESBL-EB UTIs among hospitalized patients, based on cultures collected at a single Spanish hospital, using age, gender, nursing home residence, previous antimicrobial therapy, hospitalization, and recurrent UTIs [29]. Similarly, Aviles *et al* developed a tool based on hospitalized patients at a single Chilean academic hospital with community-onset UTIs; they found that the presence of an ESBL-producing EB could be predicted based on the patient’s history of infection by ESBL-producing bacteria, recent use of antimicrobials, institutionalization or recent hospital stay, and history of metastatic cancer [30]. Of note, these prior studies only included patients who were hospitalized; their studies did not include patients who presented to outpatient offices. Thus, the results of these prior studies may not be applicable to patients being seen in an outpatient primary care practice, where the majority of UTI diagnoses are made [31].

We included patients presenting to outpatient practices in our study, and the majority were not admitted to the hospital. This is of particular importance as patients presenting to community practices or an ED are less likely to have prior microbiology data available to guide empiric treatment decisions. As a result, the clinical prediction tool developed here may be more applicable for patients presenting to an outpatient practice. In addition, our study is the first to be derived from a North American cohort, making it potentially more generalizable to other North American clinical practices that have similar rates of MDR organisms.

The proposed clinical prediction tool is likely to have significant diagnostic and therapeutic utility. It can be used to identify those patients for whom it is important to obtain a urine culture on initial presentation for uncomplicated UTI. The predictive model will also help guide empiric antibiotic therapy in patients with more severe infection while awaiting culture results. If such a patient were found to be low risk for an ESC-R EB as the etiology of their infection, empiric antibiotics with a narrower spectrum would be justified and thus promote antibiotic stewardship efforts. Implementation of this scoring system in clinical practice is expected to be of low financial burden, which is an important consideration as antibiotic stewardship strategies typically require significant financial and human resources [32]. Further studies of the economic impact of this tool, particularly related to stewardship, would be highly valuable.

There are potential limitations of our study. First, misclassification is a concern in retrospective studies and when developing a prediction tool. However, potential case and control subjects identified by the clinical microbiology laboratory were then screened by an infectious diseases-trained physician who performed standardized chart review to determine whether true infection was present, rather than using diagnostic or billing codes. Second, external validation of the prediction tool could not be performed, as the study cohort was not large enough to permit partitioning of the population for derivation and validation. The bootstrapping technique was thus used to perform internal validation, but further study is needed to externally validate this prediction tool. Third, the subjects in this study were enrolled between 6 and 9 years ago, and the rate of ESC-R EB UTIs in the community may have increased since then; however, on review of UPHS ambulatory urine cultures from 2017–2018, there was not a substantial shift in the prevalence of ESC-R among EB suggesting the results of this study remain relevant. Finally, this prediction tool was based on a single healthcare system in North America, and the performance of the prediction tool may vary based on regional rates of ESC-resistance and may not be generalizable to dissimilar patient populations.

## CONCLUSIONS

The results of our study demonstrate that community-onset UTI due to an ESC-R EB can be predicted using the proposed scoring system, which can help guide empiric antibiotic choice and urine culture ordering. Further studies are needed to validate this tool in additional populations and to assess the clinical and economic impact of its implementation.
